# The association between diabetes and nocturia: A systematic review and meta-analysis

**DOI:** 10.3389/fpubh.2022.924488

**Published:** 2022-10-03

**Authors:** Zhiwei Fu, Fang Wang, Xing Dang, Tao Zhou

**Affiliations:** ^1^Department of Pediatric Surgery, Affiliated Hospital of Chengdu University of Traditional Chinese Medicine, Chengdu, China; ^2^Department of Nutrition, Dazhou Central Hospital, Dazhou, China; ^3^Department of Pediatric Surgery, Dazhou Central Hospital, Dazhou, China

**Keywords:** nocturia, diabetes, risk, systematic review, meta-analysis

## Abstract

**Background:**

Many studies have explored the association between diabetes and nocturia, but it remains unclear. This article systematically analyses existing evidence of the relationship between diabetes and nocturia, including subgroup analysis based on the number of voids, gender, and continent, in the hope of reaching more reliable clinical conclusions relating to diabetes and nocturia.

**Methods:**

PubMed, Web of Science, and Cochrane Library were searched for identifying studies relating to diabetes and nocturia prior to July 2021. Literature quality evaluation was performed using the Newcastle Ottawa Scale. A random effect meta-analysis was used for pooled odds ratios (ORs) and confidence intervals (CIs) as a means of evaluating the relationship between diabetes and nocturia.

**Results:**

In total, 29 of 781 potentially relevant studies were proven to be eligible. The overall pooled OR demonstrated that diabetes increases the risk of nocturia (OR: 1.49; 95% CI: 1.38, 1.61; *P* < 0.00001). The association was found to be more robust among subjects ≥ 1 void than ≥ 2 void (OR: 1.74; 95% CI: 1.41, 2.14; *P* < 0.00001 vs. OR: 1.45; 95% CI: 1.33, 1.59; *P* < 0.00001), in males than females (OR: 1.59; 95% CI: 1.41, 1.79; *P* < 0.00001 vs. OR: 1.41; 95% CI: 1.20, 1.66; *P* < 0.0001) and in Asia than Europe or North America (OR: 1.54; 95% CI: 1.36, 1.75; *P* < 0.00001 vs. OR: 1.43; 95% CI: 1.19, 1.72; *P* = 0.0001 vs. OR: 1.45; 95% CI: 1.22, 1.73; *P* < 0.0001).

**Conclusions:**

Diabetes has an association with a 1.49-fold higher risk of nocturia. This association is more robust for Asian and male subjects or those at a lower nocturia cut-off.

## Introduction

Nocturia is an incredibly common and bothersome lower urinary tract symptom (1). The incidence of nocturia increases with age. Large-scale investigations have found the incidence of nocturia of ≥ 2 times per night in 60-year-old to be approximately 25% (2). In addition to sleep disruption and impaired quality of life, nocturia can also result in falls, fractures, and increased mortality among the elderly. High-quality meta-analysis has proven that nocturia increases the risk of falls by approximately 20% and that of fractures by 32% (3). In addition, another meta-analysis has demonstrated that nocturia has an association with a 1.27-fold risk of mortality (4). Therefore, identifying the risk factors of nocturia is of great importance.

Nocturia is closely related to age, but it has many influencing factors, namely, hypertension and diabetes (4). Recent studies have shown diabetes to be related to nocturia with a limited level of evidence. However, with the interference of age, gender, race, and other confounding factors, further research is required regarding whether diabetes is an independent risk factor for nocturia. Therefore, the aim of this article is to comprehensively analyze the relationship between diabetes and nocturia and reach a reliable conclusion for further guiding the clinical management of nocturia.

## Materials and methods

### Search strategy

Standard preferred reporting items for systematic reviews and meta-analysis (PRISMA) guidelines were adhered to when conducting this review. PubMed, Web of Science, and Cochrane Library were searched in order to identify studies relating to diabetes and nocturia that were published before July 2021. Search terms included: “nocturia and (diabetes or hyperglycemia).” Only articles that were published in English were included in the meta-analysis.

### Inclusion and exclusion criteria

Inclusion and exclusion criteria were utilized based on the PICOS (patient/population, intervention, control, outcome, systematic) methodology.

Inclusion criteria: Studies that investigated diabetes and nocturia; Articles that included odds ratios (ORs) and confidence intervals (CIs); All the included articles provided the definition of diabetes and nocturia.
Exclusion criteria: System reviews or case reports were excluded; Incomplete data or no OR and 95% CI were excluded; Data from repeatedly published articles was only included once.

### Data extraction and quality assessment

Two authors independently searched and screened the literature based on the established inclusion and exclusion criteria. The following data was extracted: First name of author, publication year, patient country, study design, sample size, gender, the definition of diabetes, the minimum number of voids per night, and the number of patients with nocturia. The Newcastle Ottawa Scale (NOS) was used for evaluating the quality of the included studies (5). All the aforementioned work was independently performed by two authors and any differing opinions were resolved through a discussion with a third author.

### Statistical analysis

The data was analyzed using RevMan 5.3. We pooled the OR and 95% CI to evaluate the effect of diabetes on nocturia, and *z*-test was used to assess for statistical significance. Computed values for Cochran's *Q* test were used to evaluate heterogeneity. Random effects model was performed for high heterogeneity among studies (*P* < 0.05 or *I*^2^ > 50%). Otherwise, the fixed effects model was used. A funnel plot across all studies was made for the evaluation of publication bias. Sensitivity analysis was performed through the removal of individual studies.

For accurately investigating the relationship between diabetes and nocturia, multiple subgroup analyses were conducted. Weighted ORs were pooled in different subgroups according to 1-void and 2-void, male and female, patient continent, single-factor and multi-factor analysis.

## Results

### Literature screening and quality assessment

**Figure 1:**
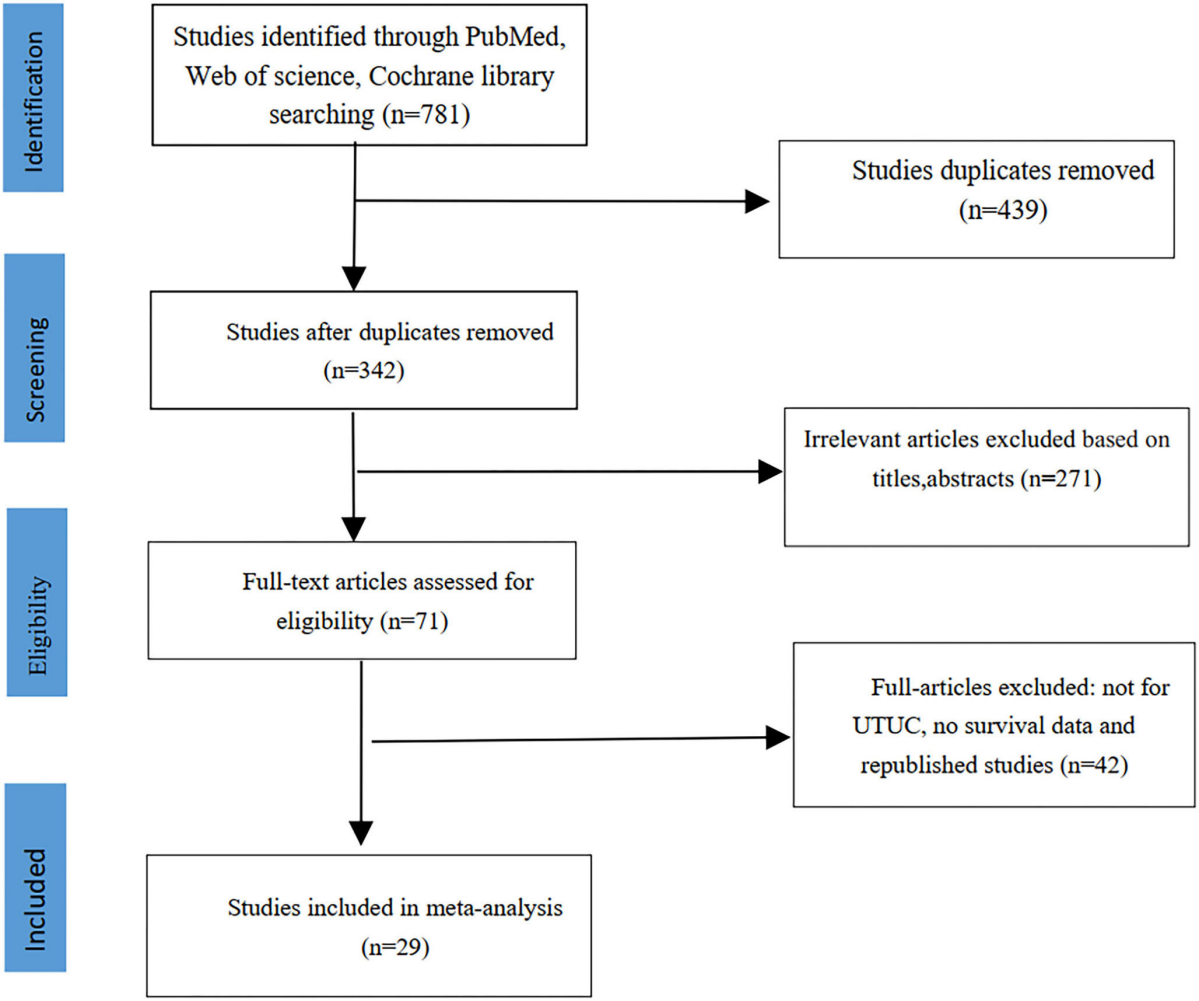
The flowchart showing study search and selection process.

We initially screened 781 abstracts, and 439 articles were deleted due to duplication. After reading the full text of 71 articles, 29 articles met the inclusion criteria and were included in this meta-analysis (6–34). The screening flowchart was shown in Figure 1.

In total, 29 articles were included in the analysis performed in this article and the quality scores of the included literature are shown in Table 1. A total of 197,809 subjects were incorporated into the meta-analysis. The basic characteristics of the literature, namely, gender, the definition of diabetes, the minimum number of voids per night, and the number of patients with nocturia, can be seen in Table 1.

**Table 1:** Basic characteristics and data of included articles.

**Study**	**Year**	**Participants country**	**Study design**	**Sample size**	**Gender (Female/ Male)**	**Diabetes definition**	**Nocturia (minimum episodes)**	**Number of nocturia patients**	**NOS**
Tikkinen et al. (28)	2009	Finnish	Questionnaires sent to subjects in the Population Register Center	3,307	N	History of diabetes	2	N	6
Liao et al. (18)	2011	Taiwan	Participants in health examinations at a Taiwan hospital	509	0/509	History of diabetes	2	N	7
Yoshimura et al. (32)	2004	Taiwan	Multistage health screening program in Taiwan	6,517	1,949/4,568	History of diabetes or fasting plasma glucose ≥ 126 mg/dL, or random glucose ≥200 mg/ dL.	2	1,856	8
Wen et al. (31)	2015	China	Multi-staged, stratified, random sampling of participants over 40 years in Zhengzhou City, China	9,637	6,621/3,016	History of diabetes	2	3,053	8
Gourova et al. (11)	2006	Netherlands	Questionnaires sent to elderly men in 21 general practices in Maastricht	2,934	0/2,934	History of diabetes	2	965	8
Hsieh et al. (12)	2008	Taiwan	Multistage selection of female participants over 60 older in Taiwan and neighboring islands	1,523	1,523/0	History of diabetes	1	1,120	8
Johnson II et al. (15)	2005	USA	Data from the Medical, Epidemiologic, and Social aspects of Aging (MESA) Study in Michigan	1,652	987/665	History of diabetes	2	520	8
Liew et al. (19)	2006	Singapore	A population-based cross-sectional survey was conducted in Singapore	2,273	1,134/1,139	History of diabetes	1	1,250	7
Obayashi et al. (24)	2015	Japan	A cross-sectional study of community-based elderly individuals	862	435/427	History of diabetes or fasting plasma glucose levels ≥ 7.0 mmol/L	2	262	8
Nakagawa et al. (23)	2010	Japan	Community sample ≥ 70 years in Japan	784	427/357	History of diabetes	2	359	7
Stone	2016	USA	Participants in USA health screening of men	30,500	0/30,500	History of diabetes	2	9,440	7
Kim et al. (16)	2018	USA	Participants aged ≥ 65 years were included from the NHANES dataset	4,698	2,323/2,375	History of diabetes or fasting plasma glucose ≥126 mg/dL, or random glucose ≥200 mg/ dL.	2	2,333	8
Azuero et al. (6)	2021	Colombia	A cross-sectional study conducted in five major cities in Colombia.	1,060	530/530	History of diabetes	1	593	7
Rembratt et al. (26)	2003	Sweden	Questionnaires sent to all inhabitants aged ≥ 65 years in Tierp, Sweden.	2,081	1,061/1,020	History of diabetes	2	603	7
Lightner et al. (20)	2012	USA	A random sample of men aged 40 – 79 years from Olmsted County, MN, USA	2,447	0/2,447	History of diabetes	2	440	7
Wang	2014	China	A cross-sectional survey for adults aged ≥18 in five geographical regions of China.	3,023	1,472/1,551	History of diabetes	2	747	8
Chow et al. (8)	2018	Taiwan	An internet-based study for subjects aged ≥ 40 years in China, South Korea, and Taiwan	8,284	4,208/4,076	History of diabetes	2	2,976	7
Huang et al. (13)	2012	Taiwan	Questionnaires sent to ≥ 40 years for the lower urinary tract symptoms in Taiwan	1,011	N	History of diabetes	2	385	7
Kim SY et al. (17)	2017	Korea	Data were collected by the Korean Centers for Disease Control and Prevention	92,626	N	History of diabetes	2	16,322	7
Madhu et al. (21)	2015	UK	A cross-sectional, population-representative survey involving 30,000 men and women from the USA, UK and Sweden evaluating lower urinary tract symptoms (LUTS)	30,000	15,810/14,107	History of diabetes	2	9,325	8
Victor et al. (29)	2019	USA	Data from a cluster-randomized trial of BP reduction in 52 black-owned barbershops in Los Angeles County, California (Clinicaltrials.gov, NCT02321618)	1,673	0/1,673	History of diabetes	2	485	7
Bing et al. (7)	2008	Denmark	Questionnaire was randomized sent to 4,000 individuals living in Copenhagen County	2,799	1,313/1,486	History of diabetes	2	1,022	8
Parthasarathy	2012	USA	Data from the Sleep Heart Health Study (SHHS) for middle-age and older adults	6,342	3,361/2,981	History of diabetes	1	3,625	7
Chung et al. (9)	2019	Korea	Data were prospective collected in Hanyang University Hospital	304	83/221	History of diabetes	2	83	7
Yow et al. (33)	2021	Malaya	A cross-sectional was conducted among community-dwelling Malaysian adults aged≥18 years old	4,616	2,634/1,982	History of diabetes	1	2,646	8
Fitzgerald	2006	USA	A multistage, stratified, cluster random sample were obtained from the Boston	5,506	3,205/2,301	History of diabetes	2	1,872	7
Zhang	2010	China	A cross-sectional survey of nocturia in several communities in northern China	1,198	592/606	History of diabetes	2	411	8
Ito et al. (14)	2019	Japan	Multiphasic health screening for 18 952 women in Fukui, Japan	18,952	0/18,952	History of diabetes	2	739	7
Mekki BS	2020	USA	A sample of 143 patients based on outpatient cardiology clinic	143	106/37	History of diabetes patients or fasting plasma glucose ≥126 mg/dL, or a recent HbA1c ≥6.5%	1	111	8

### Association of diabetes and nocturia

**Figure 2:**
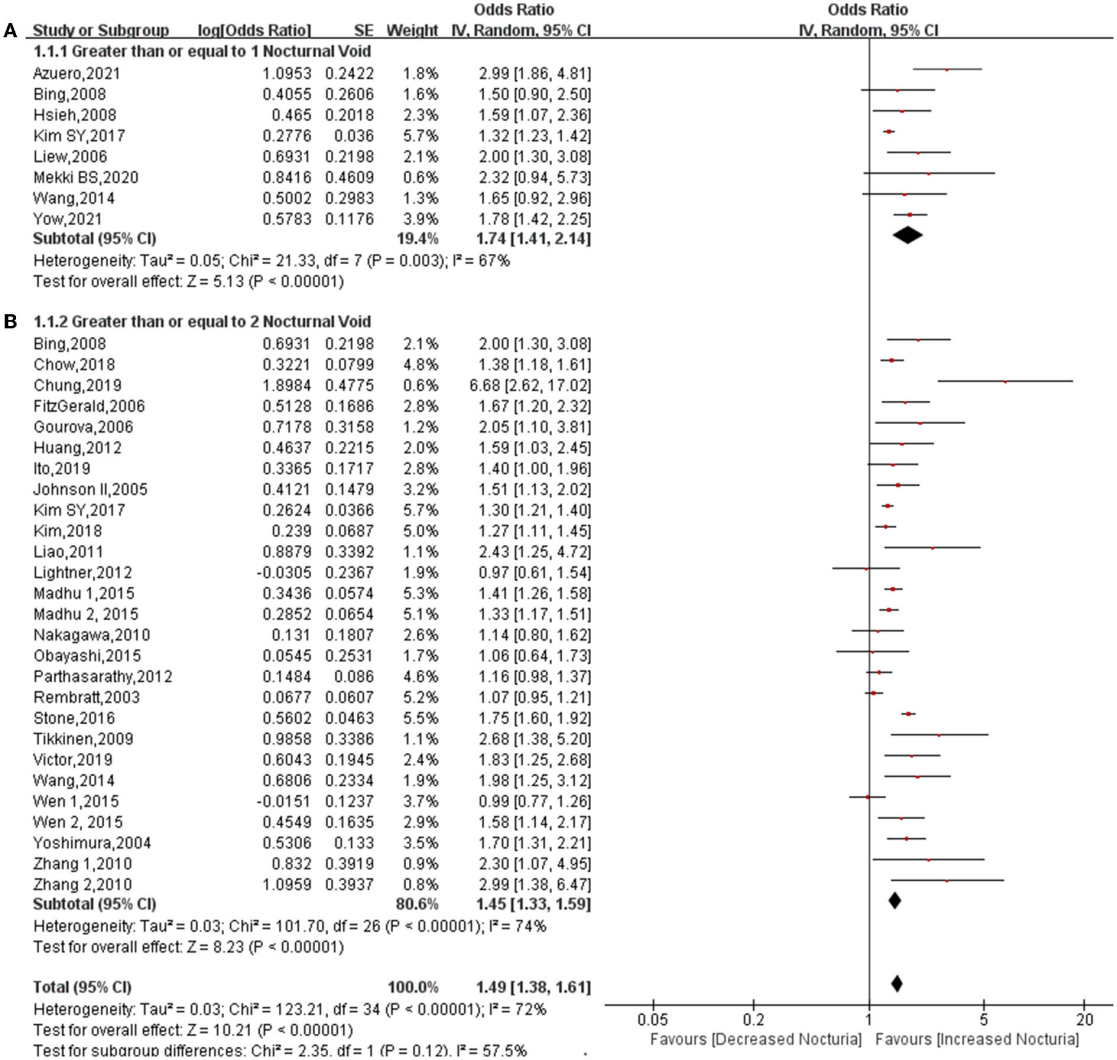
Forest plot for the association between diabetes and nocturia stratified by number of nocturia. Nocturia ≥1 void **(A)**, nocturia ≥2 void **(B)**.

All 29 studies that were included explored the association between diabetes and nocturia (6–34). The heterogeneity among studies was found to be high and the random effect model was used (*P* < 0.00001, *I*^2^ = 72%). Pooled OR demonstrated that diabetes increases the risk of nocturia (OR: 1.49; 95% CI: 1.38, 1.61; *P* < 0.00001) (Figure 2). In subgroup analysis based on the number of voids, the association was found to be more robust in subjects ≥ 1 void than ≥ 2 void (OR: 1.74; 95% CI: 1.41, 2.14; *P* < 0.00001 vs. OR: 1.45; 95% CI: 1.33, 1.59; *P* < 0.00001).

### Stratification by gender

**Figure 3:**
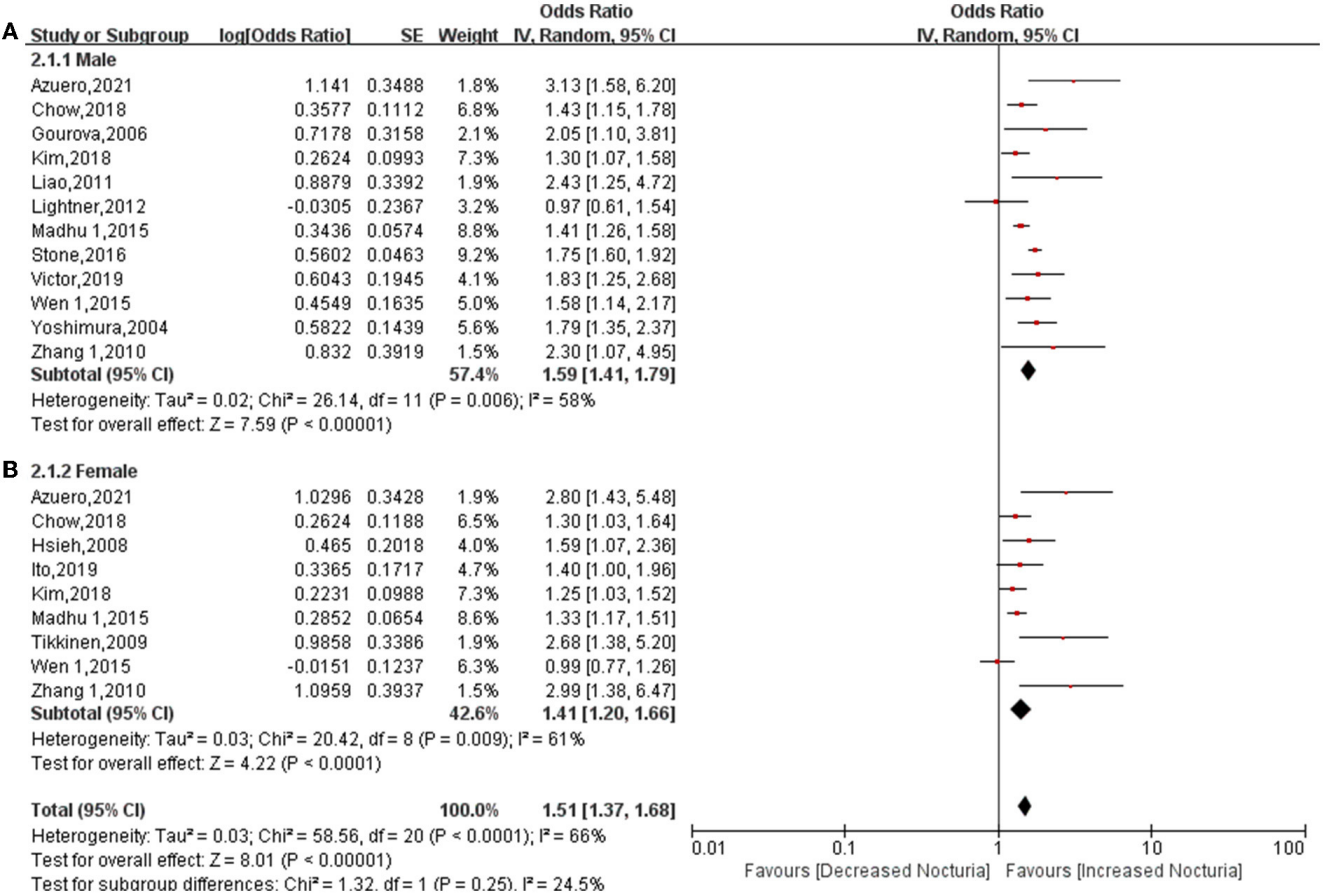
Forest plot for the association between diabetes and nocturia stratified by gender. Men **(A)**, Women **(B)**.

For subgroup classification according to gender, 12 studies provided data relating to men (6, 8, 11, 16, 18, 20, 21, 27, 29, 31, 32, 34) and nine studies provided data relating to women (6, 8, 12, 14, 16, 21, 28, 31, 34). Pooled OR showed that diabetes increases the risk of nocturia for men (OR: 1.59; 95% CI: 1.41, 1.79; *P* < 0.00001) and women (OR: 1.41; 95% CI: 1.20, 1.66; *P* < 0.0001) (Figure 3). Heterogeneity among both men (*P* = 0.006, *I*^2^ = 58%) and women (*P* = 0.009, *I*^2^ = 61%) was found to be lower than heterogeneity for the overall cohort (*P* < 0.0001, *I*^2^ = 66%).

### Stratification by country

**Figure 4:**
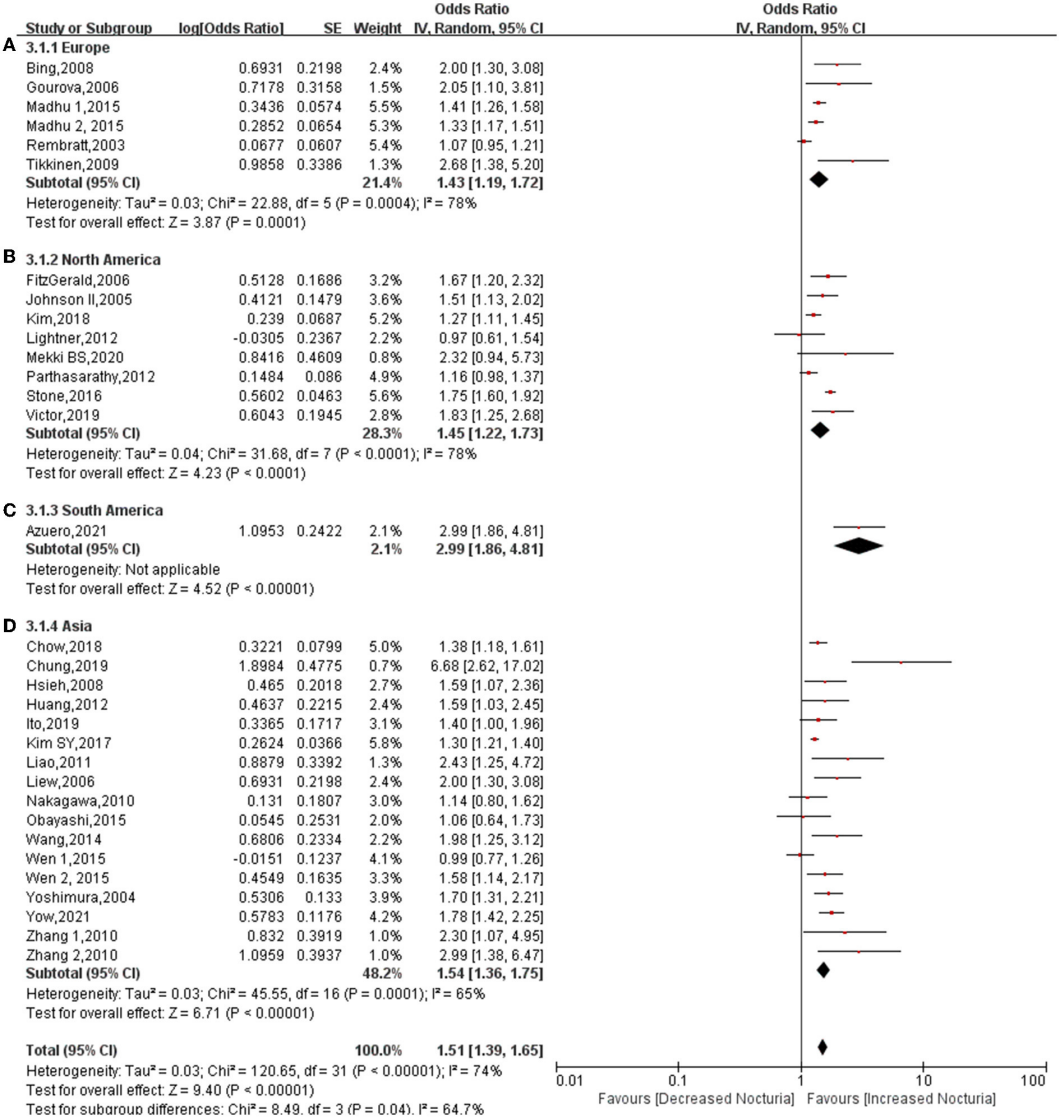
Forest plot for the association between diabetes and nocturia stratified by country. Europe **(A)**, North America **(B)**, South America **(C)**, Asia **(D)**.

In total, 5 studies provided data relating to Europe (7, 11, 21, 26, 28), 8 studies provided data relating to North America (10, 15, 16, 20, 22, 25, 27, 29), 1 study provided data relating to South America (6), and 15 studies provided data relating to Asia (8, 9, 12–14, 17–19, 23, 24, 30–34). Regardless of the continent, diabetes increases the risk of diabetes. The pooled OR for the Asia subgroup was 1.54 (95% CI: 1.36, 1.75; *P* < 0.00001). The pooled OR for Asian participants was higher than for Europe subgroup (OR: 1.43; 95% CI: 1.19, 1.72; *P* = 0.0001) or North America (OR: 1.45; 95% CI: 1.22, 1.73; *P* < 0.0001) (Figure 4). A South American study showed that diabetes increases the risk of nocturia to a greatest extent (OR: 2.99; 95% CI: 1.86, 4.81; *P* < 0.00001). Heterogeneity among both Europe (*P* = 0.0004, *I*^2^ = 78%) and North America (*P* < 0.0001, *I*^2^ = 78%) was higher than the heterogeneity for the overall cohort (*P* < 0.00001, *I*^2^ = 74%). In contrast, the heterogeneity of the Asia participants (*P* = 0.0001, *I*^2^ = 65%) was lower than the overall cohort. High heterogeneity among subgroups (*P* = 0.04, *I*^2^ = 64.7%).

### Stratification by univariate and multivariate analysis

**Figure 5:**
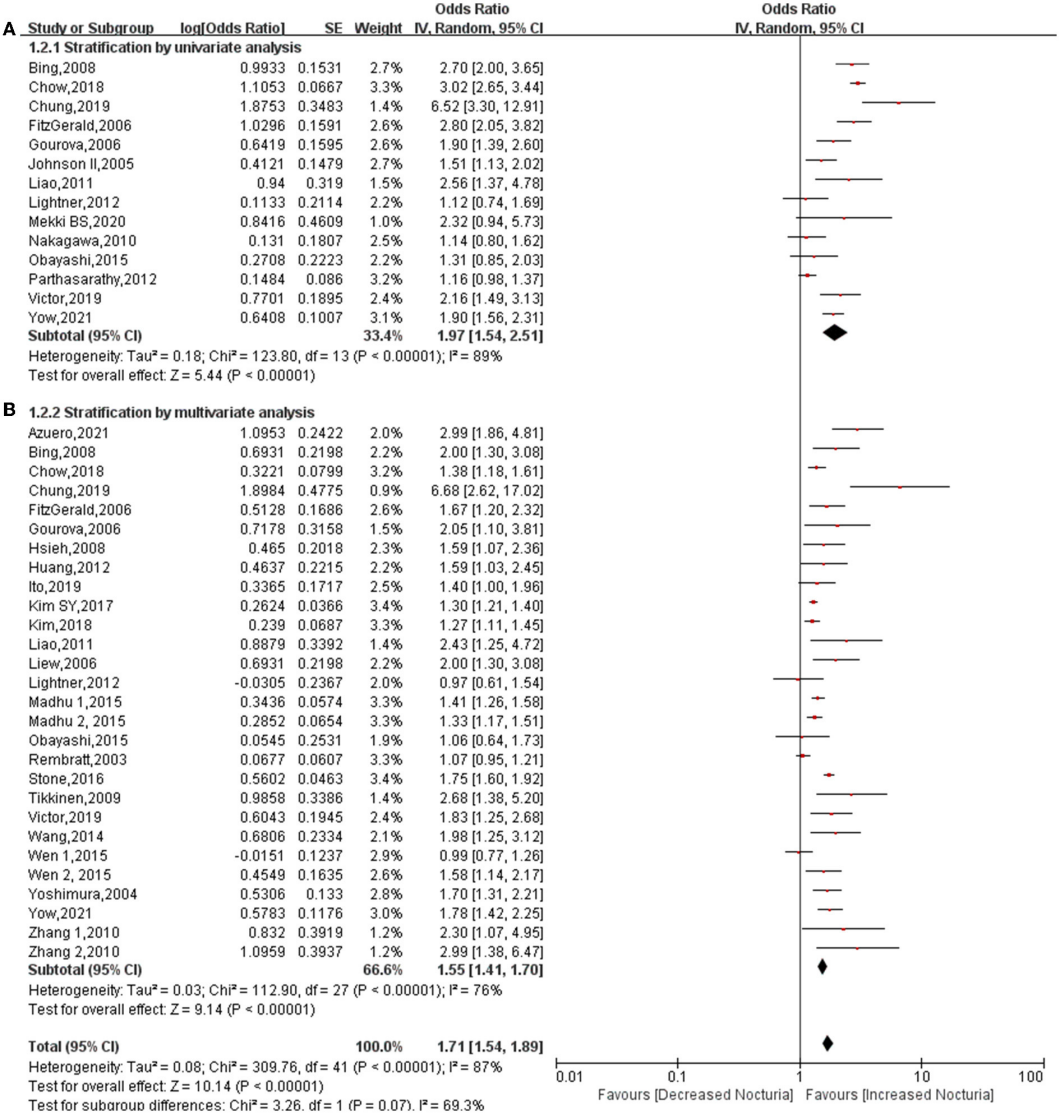
Forest plot for the association between diabetes and nocturia stratified by univariate analysis **(A)** and multivariate analysis **(B)**.

A total of 14 studies provided data relating to univariate analysis (7–11, 15, 18, 20, 22–25, 29, 33) and 25 studies provided data relating to multivariate analysis (6–14, 16–21, 24, 26–34). The pooled results proved that diabetes significantly increases the risk of nocturia in univariate analysis (OR: 1.97; 95% CI: 1.54, 2.51; *P* < 0.00001). The pooled OR for univariate was found to be higher than the overall results (OR: 1.71; 95% CI: 1.54, 1.89; *P* < 0.00001), while the pooled OR for multivariate analysis (OR: 1.55; 95% CI: 1.41, 1.70; *P* < 0.00001) was lower than the overall results (Figure 5). Heterogeneity among univariate (*P* < 0.00001, *I*^2^ = 89%), multivariate (*P* < 0.00001, *I*^2^ = 76%), and overall analysis (*P* < 0.00001, *I*^2^ = 87%) was found to be higher. The heterogeneity among subgroups was high (*P* = 0.07, *I*^2^ =69.3%). This indicates that multivariate analysis can weaken the interference other factors have on the results.

### Sensitivity analysis and publication bias

We constructed a funnel plot to detect publication bias for diabetes and nocturia frequency. There is no publication bias for all studies (Supplementary Figure 1). When performed sensitivity analysis by removing individual studies, no sources of heterogeneity were found.

## Discussion

This is believed to be the first meta-analysis that explores the relationship between diabetes and nocturia. The conclusions reached following this systematic review have significant guiding value for clinical practice. First, of the 197,809 subjects that were analyzed, diabetes increased the risk of nocturia by approximately 49%, the probability increasing to 1.74-fold for subjects ≥ 1 void nocturia. In addition, in subgroup classification based on gender, diabetes increased the risk of nocturia among males (OR: 1.59; 95% CI: 1.41, 1.79; *P* < 0.00001) and females (OR: 1.41; 95% CI: 1.20, 1.66; *P* < 0.0001). The association between diabetes and nocturia was found to be stronger in male subjects than in female subjects. In addition, the pooled OR for Asia (OR: 1.54) was found to be higher than Europe (OR: 1.43) and North America (OR: 1.45). There is a greater likelihood of diabetes being related to nocturia in Asians than in Europeans and Americans. Furthermore, diabetes significantly increased the risk of nocturia in univariate analysis (OR: 1.97), but OR dropped to 1.55 in the multivariate analysis. This demonstrates that many factors interfere with the effect of diabetes on nocturia, and these factors will be discussed later.

Most studies have found that after making adjustments for other factors, diabetes is an independent risk factor for nocturia (6, 18, 32). However, relatively few studies have reported that diabetes and nocturia are two independent diseases (20, 24). Diabetes is a common cause of nocturia for several reasons. Osmotic diuresis secondary to hyperglycemia can significantly increase the output of urine during the night (35). In addition, diabetes-induced cerebrovascular disease or peripheral nerve stimulation resulting in bladder sensory dysfunction or detrusor overactivity may be a cause of overactive bladder (36). A survey that was conducted in Japan found that 25% of patients with diabetes also had bladder detrusor hyperreflexia (37).

Although a strong correlation exists between nocturia and age, the link between diabetes and nocturia appears to have no connection with age, potentially due to the fact that the prevalence of diabetes increases with age. Many studies have found that following adjustments for the effects of age, diabetes has a significant association with nocturia (18, 32). The results of this study are in accordance with previous studies that found diabetes to increase the risk of nocturia even following adjustments made for age, gender, and other factors in multivariate analysis (OR: 1.55).

The more robust relationship between diabetes and nocturia remains controversial in men in comparison to women. In the subgroup classification based on gender in this article, the association between diabetes and nocturia was found to be slightly stronger among men (OR: 1.59) than women (OR: 1.41). However, a meta-analysis indicated that the correlation between hypertension and nocturia is stronger in women (OR: 1.45) than in men (OR: 1.28) (38). This demonstrates that the influence of gender on nocturia is interfered with by accompanying diseases. In addition, the influence gender has on nocturia and the influence diabetes has on bladder function are also interfered with by several confounding factors. In a study that was conducted by Tikinen et al. (39) women younger than 50 years were found to have a higher incidence of nocturia than men of the same age, but the increase rate of nocturia in men was observed as being twice as fast as that of older women. Bing et al. noted that although there is a similar prevalence of nocturia in men and women, women have a higher tolerance to nocturia than men (7). A study found bladder dysfunction caused by diabetes to account for 59.26% of women and 74.07% of men (40). This proves that the degree of association between diabetes and nocturia differs between genders.

Many studies have reported the incidence of nocturia to vary between people of different races. Limited by several races included in one study, subgroup analysis could only be performed by continent. Only one study in South America has investigated the relationship between diabetes and nocturia, so the level of evidence for the results is incredibly low. A more robust association was found between diabetes and nocturia in Asia (OR: 1.54) than in Europe (OR: 1.43) or North America (OR: 1.45). The strong association between diabetes and nocturia in Asia may prove to be particularly useful, particularly considering the fact that Asians are more prone to organ damage resulting from diabetes. The inclusion of several races in individual studies has resulted in particularly high heterogeneity within the group. Therefore, the correlation between diabetes and nocturia in different continents warrants further study.

The strengths of this review include a contemporary search of studies published in English, duplicate assessment of inclusion criteria, and the quality of evidence and extracted data. This is believed to be the first meta-analysis that explores the relationship between diabetes and nocturia. Through an overall evaluation and subgroup analysis, the results of this study provide evidence of an association between diabetes and nocturia. However, there were inevitably some limitations with the meta-analysis in this study. First, many subjects were diagnosed with diabetes based on their medical histories rather than the current status of hyperglycemia. Second, the nocturia data that was obtained through questionnaires was found to be too subjective. Third, pertinent diseases such as hypertension, obesity, and other diseases that may strengthen the association between diabetes and nocturia were not examined. Fourth, the subgroup analysis by country was unable to provide an analysis based on race. At last, the significant difference in the number of cases that were included in the study may lead to biased results.

## Conclusions

Diabetes has an association with a 1.49-fold higher risk of nocturia. This association is more robust for Asian and male subjects or those at a lower nocturia cut-off.

## Data availability statement

The original contributions presented in the study are included in the article/Supplementary material, further inquiries can be directed to the corresponding author.

## Author contributions

ZF wrote the manuscript. FW collected and analyzed the data. XD helped the review and revised the manuscript. TZ helped to design the study and revised the article. All authors have read and approved the manuscript.

## Conflict of interest

The authors declare that the research was conducted in the absence of any commercial or financial relationships that could be construed as a potential conflict of interest.

## Publisher's note

All claims expressed in this article are solely those of the authors and do not necessarily represent those of their affiliated organizations, or those of the publisher, the editors and the reviewers. Any product that may be evaluated in this article, or claim that may be made by its manufacturer, is not guaranteed or endorsed by the publisher.
