# Supplementary figures and images for: The association between diabetes and nocturia: A systematic review and meta-analysis
Source: Front Public Health. 2022 Oct 3;10:924488. doi: 10.3389/fpubh.2022.924488 (PMC9574227; doi:10.3389/fpubh.2022.924488)

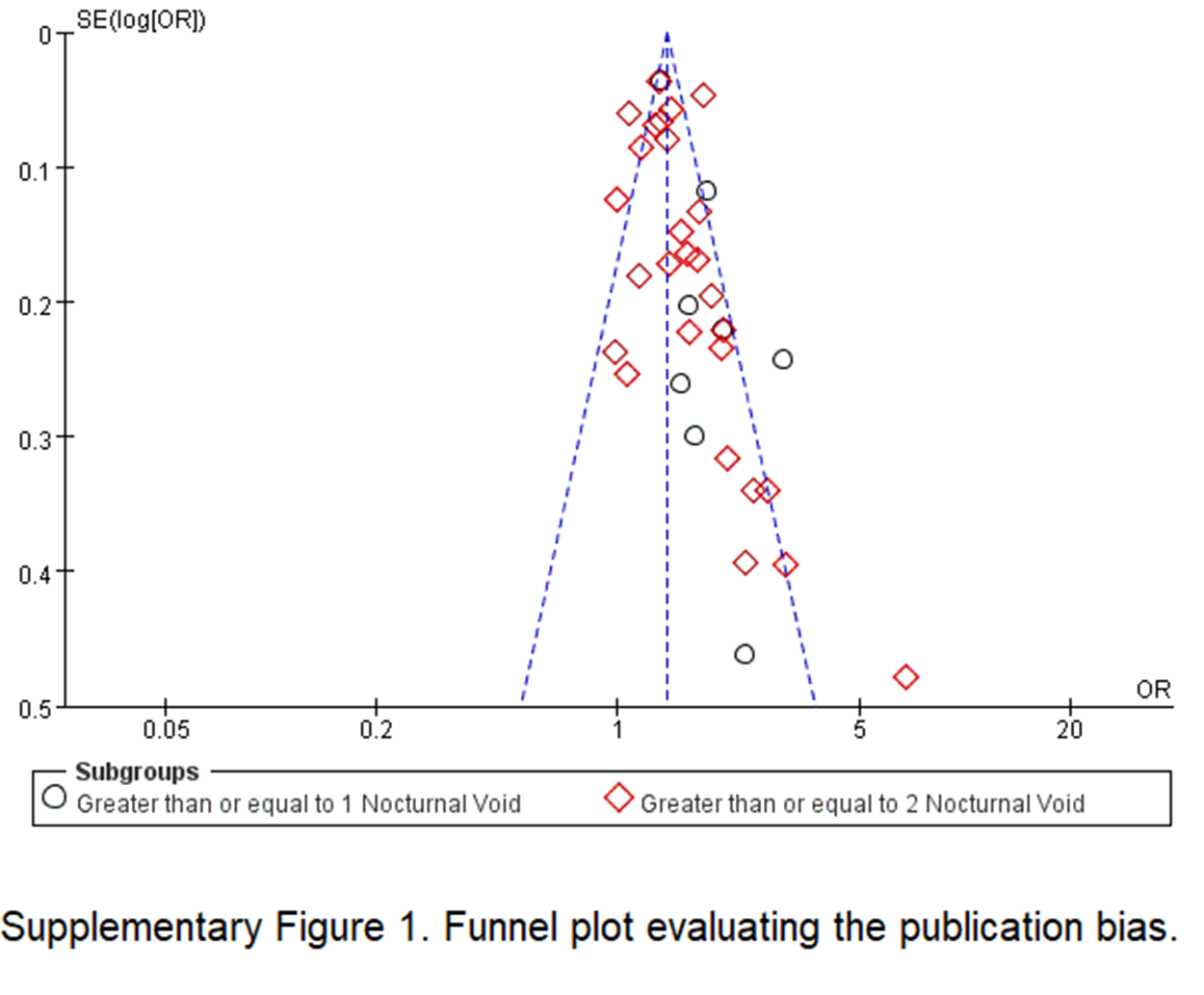

Supplement: Supplementary file 1 [file Image_1.TIF]
